# Early-Life Trauma as a Trigger for Developmental Myofascial Dysfunction: A Case Report

**DOI:** 10.7759/cureus.87863

**Published:** 2025-07-13

**Authors:** Gillian R Lauder, Jessica Luo, Judith Nassaazi

**Affiliations:** 1 Department of Anesthesiology, Pharmacology and Therapeutics, University of British Columbia, Vancouver, CAN; 2 Department of Pediatric Anesthesia, British Columbia Children's Hospital, Vancouver, CAN; 3 Department of Pediatric Anesthesia, British Columbia Children's Hospital Research Institute, Vancouver, CAN; 4 Faculty of Science, University of British Columbia, Vancouver, CAN; 5 Department of Orthopaedics, CoRSU Rehabilitation Hospital, Kisubi, UGA

**Keywords:** chronic pain, developmental dysplasia, myoactivation, myofascial dysfunction, orthopedic, pediatric, trigger point injections

## Abstract

Skeletal asymmetry is a major, unrecognized contributor to myofascial dysfunction and chronic pain in children and adolescents. myoActivation® is a novel medical intervention for the assessment of skeletal asymmetries and treatment of myofascial chronic pain. The therapeutic component of myoActivation involves a needling technique to release myofascial trigger points and scars. myoActivation restores normal mechanics, reduces pain, and improves balance. We report the case of an 18-month-old boy with a history of an asymmetrical gait and a diagnosis of mild developmental dysplasia of the hip, identified following a minor injury. He received trigger point injections to the muscles around his pelvis using myoActivation principles. An immediate improvement in symmetry was observed, resulting in improved gait, without a limp, and was also associated with a change in his hip X-rays. Based on these observations, myoActivation may be an effective assessment and therapeutic tool for other children with developmental myofascial dysfunction.

## Introduction

Developmental dysplasia of the hip (DDH) represents a spectrum of developmental abnormalities that range from minor laxity of ligaments to complete luxation [1,2]. DDH is the most common orthopedic disorder in newborns [3]. For infants older than 4-6 months, pelvis radiography is utilized to confirm the diagnosis. However, even mild forms of DDH, in which there are subnormal patterns of acetabular shape and coverage, may predispose children to mechanical dysfunction and instability [4]. DDH is a three-dimensional deformity of the acetabulum (confirmed by computerized tomography scan), and instability may exist with only minor changes on two-dimensional imaging [5].

There is limited understanding of how joints take on their range of distinctive shapes or joint morphogenesis [6]. Hip joint development and maturation is a dynamic process influenced by genetics, environmental factors, and the mechanical loading equilibrium of the joint [1,7]. Humans exhibit biotensegrity, whereby the body functions as a three-dimensional visco-elastic entity no matter what position it adopts [8]. The biotensegrity model dictates that each individual part of the organism combines with the mechanical system to create an integrated functional movement unit to achieve skeletal symmetry, balance, coordination, posture, and locomotion. With this model in mind, it is understandable that reduced or restricted movement, torsional deformities, and unloaded hip joints have been identified as risk factors for DDH [6,7,9].

myoActivation is a structured process that synthesizes aspects of the history, postural analysis, and examination with the results of systemized movement tests to target the most likely tissues responsible for the presenting myofascial dysfunction and pain [10]. The myoActivation assessment process relies on recognizing the importance of the timeline of lifetime trauma and the mechanisms of each injury. The earliest or most significant physical trauma in childhood is often an inciting event for the development of myofascial dysfunction and subsequent chronic myofascial pain [11]. The history should capture any motor vehicle accidents, falls, fractures, major and minor surgeries, and scars as well as the consequences and healing process of these traumatic events [12].

The therapeutic component of myoActivation is a needling technique. Needling targets palpable pain points (PPPs) in muscle (muscle trigger points (MTrPs)) and fascia (fascial trigger points (FTrPs)), as well as scar tissue, and skin creases. Needling with a hollow-bore cutting tip needle at MTrPs triggers muscle activation, which is the restoration of a muscle from a state of sustained contraction to its normal relaxed condition. The result is instant, with sustained improvement in pain [13], flexibility, fluidity of movements, and range of motion [14]. Needling these tissues restores normal mechanics and symmetry. Recent papers have described this technique and its potential efficacy in chronic pain [10-12], including pain relief in adolescent patients [13,14].

## Case presentation

**Figure 1:**
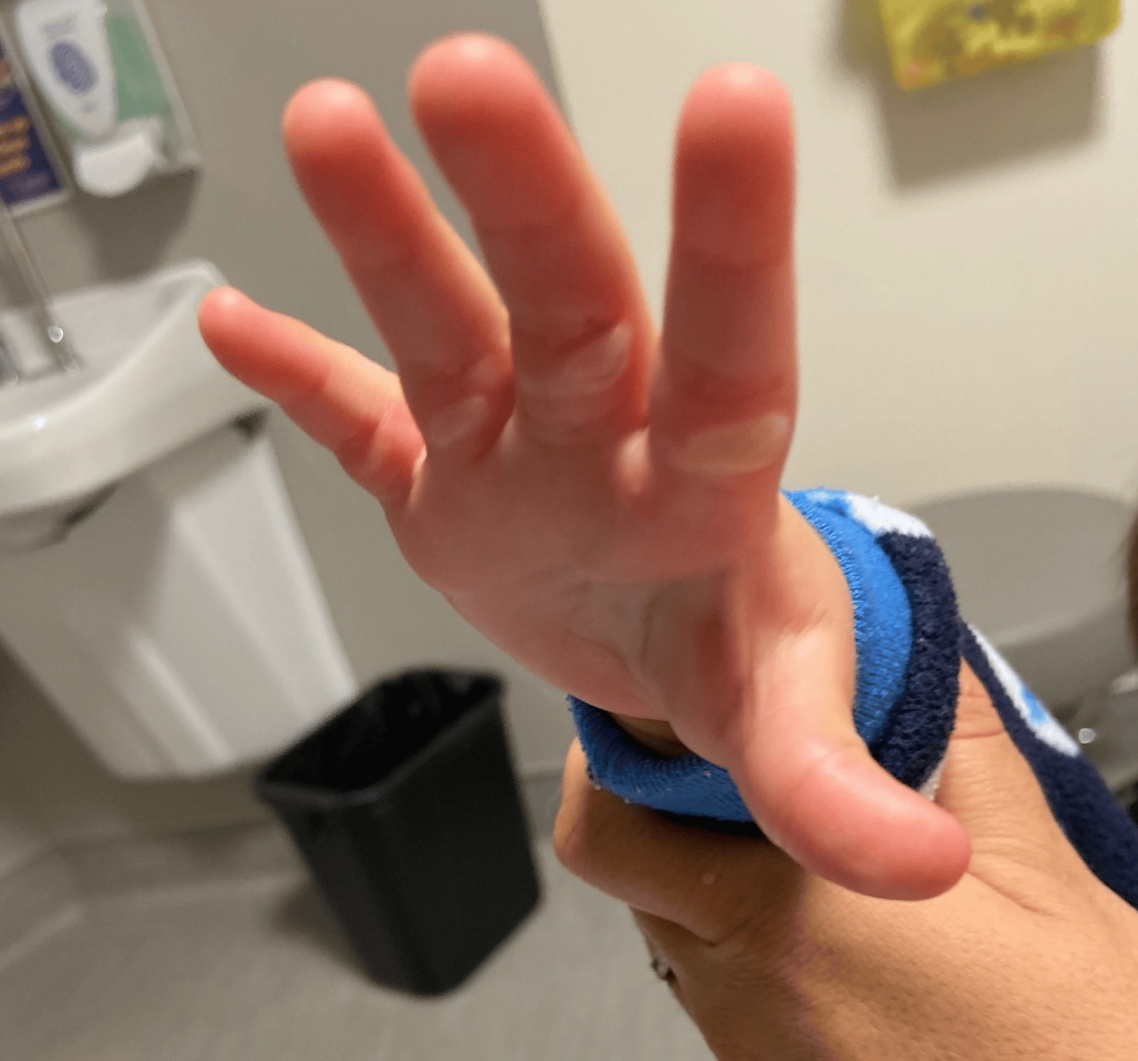
Burn injury on hand. Resulting blisters were treated appropriately, but the rotational movement at the time of this injury may have contributed to subsequent asymmetries in the infant's posture while crawling.

An 18-month-old boy, born at term by normal spontaneous vaginal delivery, was referred to the interventional team of the Complex Pain Service (CPS) at British Columbia Children's Hospital (BCCH) for the assessment and management of skeletal asymmetries. He had had no developmental concerns until one year of age, one month following a burn injury to his right hand. This burn resulted from the infant reaching into a baseboard heater. His hand was stuck in the grid system of this heater, and he did a rotational movement of his torso to pull his hand away from the heater. He sustained blisters from that burn (Figure 1), which were treated appropriately in the emergency room and followed on with a cast for three weeks and repeat dressing changes to promote healing. In the first week after the cast was removed, the boy crawled using both hands, on the left on a flattened palm, but using a fist with the right. He started to walk at 14 months. At this time, his father, a physiotherapist, noted several asymmetries in his posture and locomotion: he reported clicks in the right hip and a preference for standing on the left side over the right.

Prior to CPS referral, the patient was seen by a pediatrician and the BCCH orthopedic team. The pediatrician noted slight right DDH. The orthopedic clinical examination confirmed mild right DDH and was reported to be not clinically significant; however, a full examination was challenging due to the child's lack of cooperation. This toddler had almost none of the classic risk factors associated with DDH: he was not a first-born child, pregnancy was not associated with oligohydramnios or breech presentation, and the patient was not female. However, he did have a family history of DDH; his paternal grandfather had DDH. In this case, we propose that myofascial dysfunction may have contributed to the observed presentation of mild DDH resulting from minor trauma.

When he was seen by the CPS interventional team, a complete myoActivation assessment examination could not be performed given his age, level of cooperation, and inability to follow commands for specific tasks. At the time of presentation to CPS, there was no visible scarring at the site of his burn. However, he appeared to have PPPs centered on the right vastus lateralis, right rectus femoris, and left quadratus lumborum. He also had a torso shift to the left.

He was started on supplements (magnesium bisglycinate/vitamin D3 and vitamin K2), appropriate for his weight, to help relax muscles in sustained contraction. His parents were advised to gently massage areas of PPPs.

At the one-month follow-up appointment, his parents had not noticed much improvement in the previously reported asymmetries; however, they had had some issues getting a formulation of magnesium that was palatable for their son, so he was not on the recommended dose. At that time, it was decided to continue with conservative management with the aim of achieving an adequate dose of magnesium.

At the four-month follow-up, his parents reported that the torso shift had improved. Right leg issues at this time included external rotation of the leg when he sat on his three-wheeler bike, only rolling to the left when he was lying on his abdomen, not being happy to straighten his hip, and not liking any massage to the upper thigh muscles. myoActivation was discussed as a means of releasing the remaining muscles in sustained contraction.

Investigations

**Figure 2:**
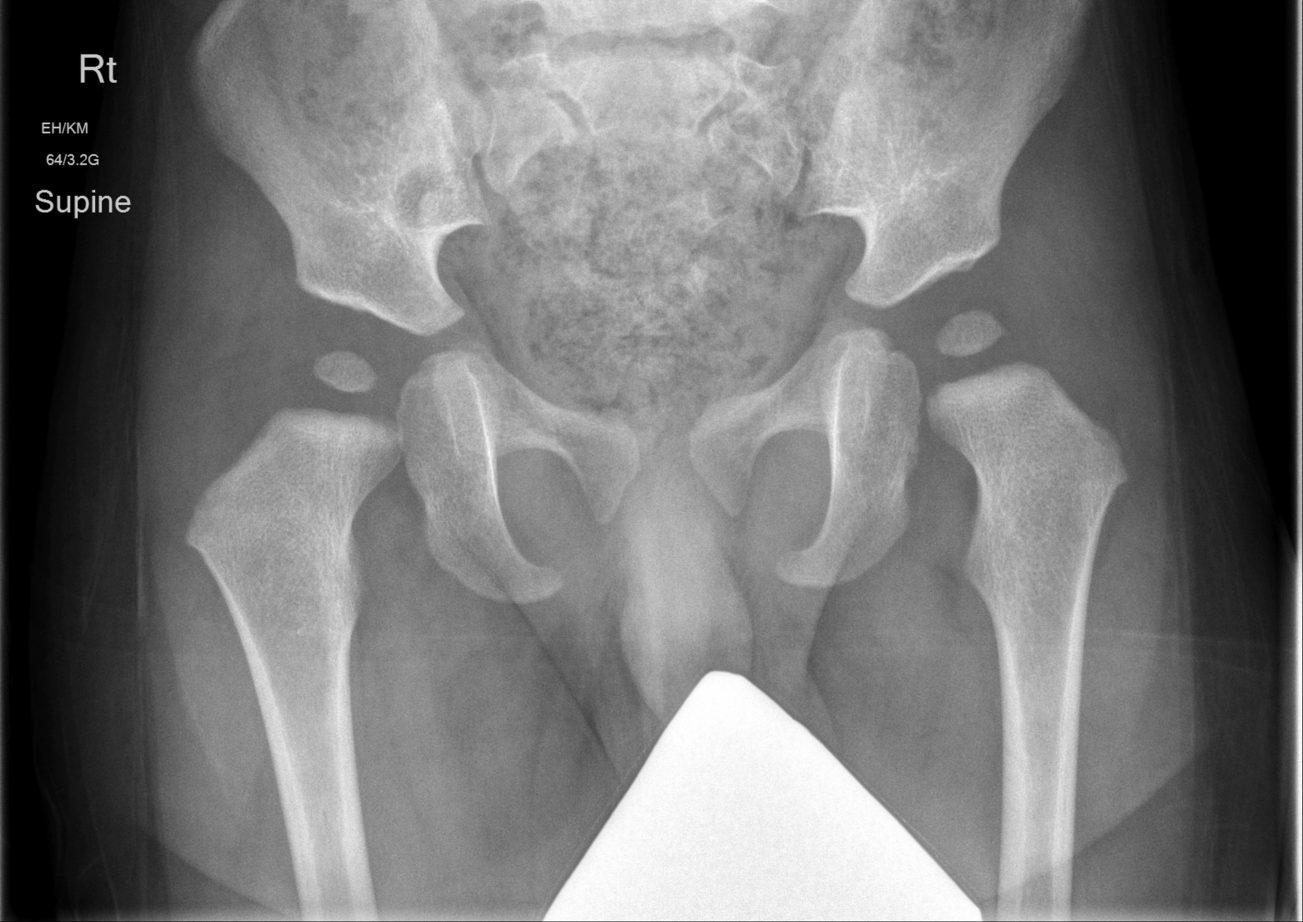
Pre-intervention pelvic X-ray. Acetabular index was 27.5° on the right and 28.4° on the left. Rt: right

**Figure 3:**
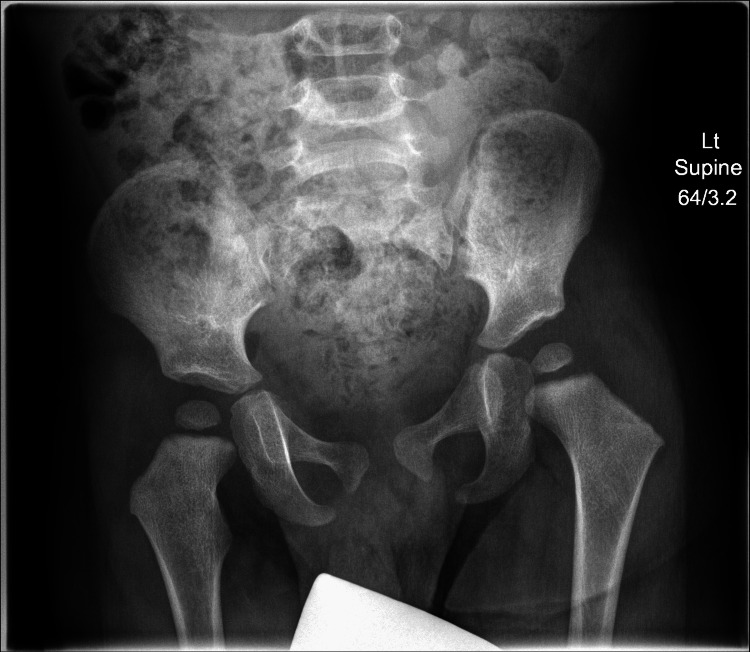
Post-intervention pelvic X-ray. Acetabular index was 25° on the right and 23.1° on the left. Lt: left

Radiological evaluation for DDH for this child's age group relies on assessing the congruity of the hip joint and the acetabular index of the pelvic X-ray. In this case, the acetabular index pre-intervention was 27.5° on the right and 28.4° on the left side (Figure 2); the post-intervention acetabular index was 25° on the right and 23.1° on the left side (Figure 3). These measurements suggest that the coverage of the femoral head by the acetabulum has improved. An acetabular index angle below 25° is considered normal at 24 months [15]. Hence, while the pre-intervention measurements may have prompted clinical concern, the post-intervention measurements indicated that conventional therapeutic intervention was no longer required.

Treatment

myoActivation was performed as a day-case procedure under monitored procedural sedation when the child was 23 months old, five months after initial presentation. When sedated, he was observed to have his right leg shorter and positioned in external rotation, with hip abduction and flexion, and his right knee more flexed compared to the left. A full assessment to determine MTrPs in pelvic and leg musculature was performed under sedation. The right rectus femoris, right iliopsoas, right vastus lateralis, and left quadratus lumborum were activated. There were no complications at the time of injections, and the boy was discharged with the advice to use over-the-counter paracetamol and ibuprofen as required for any minor muscle discomfort.

Outcome and follow-up

Two days after myoActivation, his parents e-mailed the CPS to say that they had noticed that the click that they previously felt when they used to flex his hip to change his diaper/nappy was no longer present and that he seemed to be standing more stably, as well as putting more weight on the right side.

Five weeks after myoActivation, he was reviewed by his orthopaedic team who reported no limp, full range of movement of the hips with symmetrical abduction, and flexion up to 80°, with no leg length discrepancy (Figure 3).

At follow-up, seven months after the initial CPS assessment and six weeks after his myoActivation, his parents reported that their son was markedly improved. He was able to jump, walk up and down stairs without holding onto the handrail, push off in gait with his right leg, crouch down and kneel on both knees symmetrically, and do a lot more walking. Whereas he used to lean to the left when sitting, he was now able to sit up straight. He was not taking any over-the-counter simple analgesics. At this time, the CPS team recommended reducing the recommended supplements to a half dose.

At 12 months after initial CPS assessment and five months after his myoActivation, the boy was followed up by the BCCH orthopedic team. X-ray examination at this time showed a right acetabular index of 22° on the right and 21° on the left, suggesting sustained improvements after myoActivation treatment.

**Figure 4:**
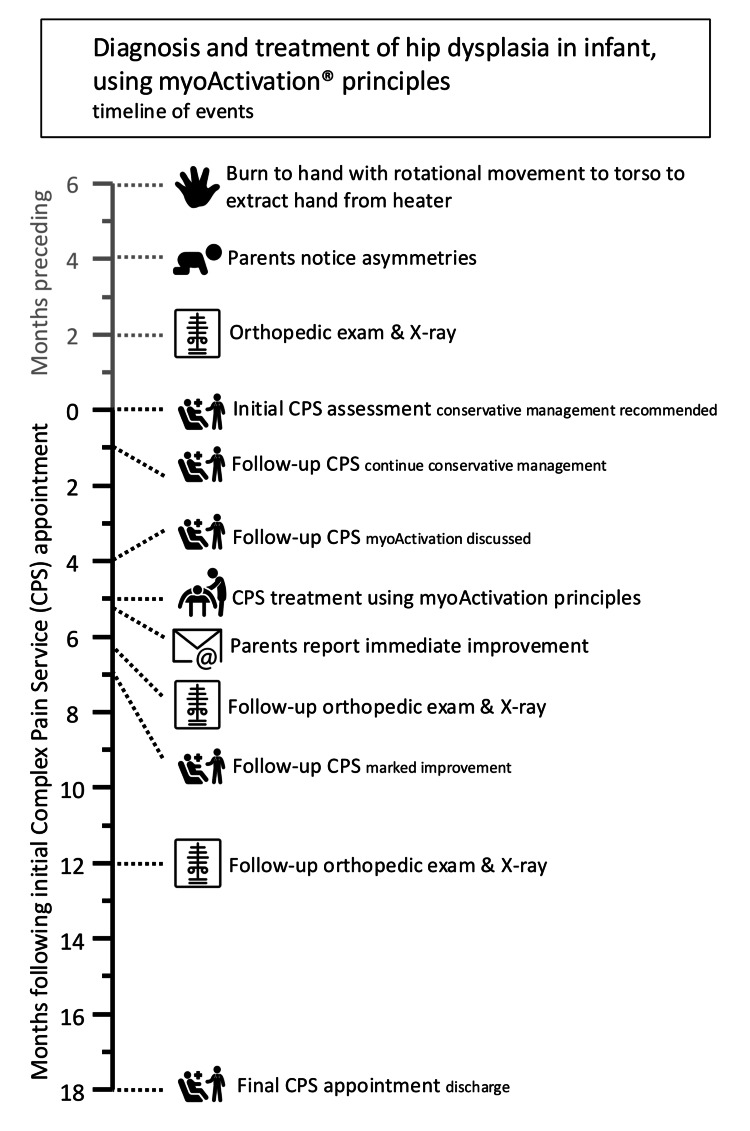
Timeline of events in the diagnosis and treatment using myoActivation principles. CPS: Complex Pain Service

At follow-up, 13 months after his myoActivation procedure, he was discharged from the care of the CPS with the recommendation to stop all supplements (Figure 4). He had no pain, had no asymmetries, and was able and willing to walk, climb, and scooter balanced on his right leg.

Parent's perspective

Pre-intervention

"When our son started to walk, he had a 'crab-legged' walk; then when he was about 15 months old, we noticed that he would stand with his upper body shifted to the left and stood with his right knee bent and hip flexed. It was very obvious when he would stand in the bathtub (without clothes on) or when he lay on the change table as he would default to this position. When sitting, he would always lean to the left, away from the right hip. While lying on his back, he would shy away from straightening his right leg and would usually prefer a flexed hip and externally rotated leg (with a flexed knee). There was an obvious palpable tight muscle in his right upper leg that was clearly tender to touch and a few tender spots in the lower back. Walking and running were asymmetric, and he would swing his right leg around. His upper body would sway significantly more left that right while running and walking. Walking stairs was hard for [him], and he would avoid walking stairs when he could and mostly wanted to be carried. He did not jump before the procedure. When outside, he mostly wanted to be carried if a walk was more than a few minutes."

Post-intervention

"Immediately (day of intervention), our son stood and walked better, no more shift and not swinging his right leg anymore. He was able to extend his right hip and knee while standing, and his thorax was more in midline as opposed to shifted left. Slowly, over the next couple of days to two weeks, he wanted to walk and run more. We also noticed this was more symmetrical. In standing, his significant left shift of the lower thorax was reduced to a minimum. Our son wants to walk himself now. He sometimes wants to sit on his dad's neck but this seems more because he enjoys it. He started jumping the day after the intervention and hasn't stopped since."

## Discussion

The paper demonstrates the case of a toddler who developed myofascial dysfunction one month after minor trauma. If left untreated, this little boy would probably have gone on to develop worsening acetabular dysplasia with or without hip subluxation. In the event that the signs and symptoms worsened, he would have needed either non-operative treatment with abduction braces or surgical intervention with either closed or open reduction of the hip joint and spica casting for over three months. In this case, myoActivation may have prevented the need for surgical intervention, sustained alteration in gait, and hip osteoarthritis. Although there was some improvement with supplements designed to relax muscles in sustained contraction, there was definite and sustained improvement in ambulating immediately following myoActivation treatment.

The exact aetiology of myofascial dysfunction is unclear. However, it is thought that muscle trauma, skeletal asymmetry, repetitive muscle stress, inflammation, and metabolic disturbances can all contribute [16]. Myofascial dysfunction and pain are associated with MTrPs, muscles in sustained contraction, and PPPs. An MTrP is a localized, hyperirritable nodule in palpable, taut skeletal muscle fibers. The presence of MTrPs is associated with muscle tenderness, weakness, and functional limitation [17-19].

The term "idiopathic" denotes any disease or condition that arises spontaneously or for which the cause is unknown. This case presented with idiopathic DDH, but it could be argued that myofascial dysfunction secondary to an injury precipitated this event. The rotational pull to extract his hand from the heater may have triggered muscles to be in sustained contraction that disrupted the biotensegrity of the child's body. Could the same principle be applied to other developmental idiopathic conditions, such as growing pains, idiopathic torsional deformities, patellar malalignment syndrome, or adolescent idiopathic scoliosis? Timing would seem to be important; early intervention may prevent progression of the myofascial dysfunction to developmental disease. Hence, early-life trauma may be the trigger or signal to pursue evidence of skeletal asymmetries.

This is the first case report of myoActivation in a toddler. It is difficult to assess myofascial dysfunction in a young, non-verbal, and uncooperative patient. They may not be able to participate fully in a myoActivation examination. Therefore, there is a heavy reliance on parent and carer observations, as well as clinical experience and acumen, to determine if there is myofascial dysfunction and to target the culprit tissue appropriately. myoActivation targets the MTrPs, FTrPs, and scars, which may all contribute to myofascial dysfunction. myoActivation treatment results in instant and maintained improvement in pain [13], flexibility, fluidity of movements, and range of motion [14]. This may not only have aetiological importance but may prevent progression to other treatment modalities, for example, prevention of progression to open reduction for DDH resistant to conservative treatment.

This is a paradigm shift in the pathophysiological conceptualization of dysfunction as it relates to idiopathic developmental myofascial disorders. It opens up a possible new understanding of pathophysiology and therapeutic approaches. It will also completely change our views of the importance of past innocent injuries in anatomically distinct areas. Only a limited amount of training would be involved in being able to notice these small asymmetry changes in toddlers and preschool children.

It will be important to establish which pediatric patient populations labelled with an idiopathic myofascial dysfunction would benefit the most from this technique and, very importantly, when it should be applied. Future studies are required to establish the efficacy of this unique practice to improve skeletal symmetry and prevent ongoing progressive myofascial dysfunction.

## Conclusions

Minor injuries are common in children. As such, developmental myofascial dysfunction may occur secondary to even minor trauma. Skeletal symmetry may be a key principle in the recognition and prevention of developmental myofascial dysfunction. myoActivation principles can be used to help diagnose myofascial dysfunction, and myoActivation therapy may help restore skeletal symmetry and function. These lessons may suggest a potential paradigm shift in the pathophysiological conceptualization of dysfunction as it relates to idiopathic developmental myofascial disorders.
